# Role of Histone Deacetylases in the Pathogenesis of Salivary Gland Tumors and Therapeutic Targeting Options

**DOI:** 10.3390/ijms241210038

**Published:** 2023-06-12

**Authors:** Maria Manou, Dimitrios S. Kanakoglou, Theodoros Loupis, Dimitrios M. Vrachnos, Stamatios Theocharis, Athanasios G. Papavassiliou, Christina Piperi

**Affiliations:** 1 Department of Biological Chemistry, Medical School, National and Kapodistrian University of Athens, 11527 Athens, Greece; maria_manou@hotmail.com (M.M.); kanakogloud@biol.uoa.gr (D.S.K.); 2 Haematology Research Laboratory, Clinical, Experimental Surgery and Translational Research Center, Biomedical Research Foundation, Academy of Athens, 11527 Athens, Greece; tloupis@bioacademy.gr (T.L.); dimvrachnos@gmail.com (D.M.V.); 3 First Department of Pathology, Medical School, National and Kapodistrian University of Athens, 11527 Athens, Greece; stamtheo@med.uoa.gr

**Keywords:** HDAC, HDAC inhibitors, epigenetic modifications, histone acetylation, histone deacetylation, salivary gland tumors, mucoepidermoid carcinoma, adenoid cystic carcinoma

## Abstract

Salivary gland tumors (SGTs) comprise a rare and heterogenous category of benign/malignant neoplasms with progressively increasing knowledge of the molecular mechanisms underpinning their pathogenesis, poor prognosis, and therapeutic treatment efficacy. Emerging data are pointing toward an interplay of genetic and epigenetic factors contributing to their heterogeneity and diverse clinical phenotypes. Post-translational histone modifications such as histone acetylation/deacetylation have been shown to actively participate in the pathobiology of SGTs, further suggesting that histone deacetylating factors (HDACs), selective or pan-HDAC inhibitors (HDACis), might present effective treatment options for these neoplasms. Herein, we describe the molecular and epigenetic mechanisms underlying the pathology of the different types of SGTs, focusing on histone acetylation/deacetylation effects on gene expression as well as the progress of HDACis in SGT therapy and the current status of relevant clinical trials.

## 1. Introduction

Salivary gland tumors (SGTs) are rare neoplasms, representing 3–6% of head and neck (HN) tumors and constituting a wide range of subtypes (Table 1) [1,2]. Overall, 80% of SGTs fall into the category of benign tumors [1,2]. Pleomorphic adenoma (PA) is a frequent benign SGT regardless of the site of origin with incidence of 60–80%, which is followed by Warthin’s tumor (WT) (papillary cystadenolymphoma), basal cell adenoma (BCA), and myoepithelioma (MYE) [2,3,4]. The benign/malignant SGT ratio is 3:1 counting 55,000 cases per year [5]. Malignant cases usually arise in parotid. However, most sublingual gland tumors, and almost 50% of SGTs of the minor glands are also malignant [1,2,3,4,5,6,7]. Around 10% of SGTs arise in the submandibular gland, and 50% of them are diagnosed as PAs [1,2,3,4,5,6]. Mucoepidermoid carcinoma (MEC) refers to the most frequent malignant SGT with a reported incidence of 30% of all malignant SGTs. However, a small number of studies report that adenoid cystic carcinoma (AdCC) is more common [3,4]. These tumors are followed by acinic cell carcinoma (AciCC) and polymorphous adenocarcinoma (PAC) along with adenocarcinomas not-otherwise specified (AC-NOS) as the most frequent malignant SGTs.

Benign SGTs show predilection for the 4th–6th decade of life, whereas most malignant SGTs occur in the 6th–7th decade. Females are more prone to PAs than males, whilst the opposite occurs for MEC. Opposed to adults, acinic cell carcinoma remains the second most frequent aggressive SGT after MEC in children [2,3,4].

The symptoms of malignant tumors include painless swelling or facial nerve paralysis [5,6], erythema and dysesthesia [8], or fluctuant masses accompanied by pain, numbness, and ulcerations of the swelling [9]. Pre-treatment magnetic resonance imaging (MRI) is the first choice toward identifying SGTs and their features (localization/anatomical spread) [10], which is followed by computed tomography and ultrasonography. Recent MRI instrumentation has been helpful in differentiating benign from malignant tumors [11].

The 5th WHO classification encompasses “mucinous adenocarcinoma”, which is characterized by AKT1 (E17K) mutations and histological heterogeneity indicating intraductal papillary mucinous neoplasms of salivary glands. Newly added malignancies enfold “sclerosing microcystic adenocarcinoma” as well as “microsecretory adenocarcinoma” [7].

Most malignant SGTs, except for salivary duct carcinomas (SDC), are considered low-grade malignancies. High-grade transformation is characterized by architectural and cytologic changes, most prominently necrosis, loss of classic architectural patterns such as duct formation, increased mitotic rate and anaplastic cells pleomorphic nuclei with enlarged nucleoli [5,12].

Recurrence is not uncommon in SGTs, with metastatic events being usually grade-related and occurring in almost half of the cases (25–50%) [13]. High-grade neoplasms are linked to high morbidity due to distant metastases via hematogenous spread. Tumors such as AdCC and SDC display distant metastases in lung, liver, or bone [14] with the 5-year survival ratio of relapsed SGTs being just about 20% [13].

Relapse might be preventable, but appropriate therapy requires risk determination, which is conditional to factors including clinical manifestation, imaging, histologic type-characteristic and molecular biomarkers. Primary radiotherapy is employed for inoperable tumors, and adjuvant radiotherapy is selected in extensive nodal and/or locoregional involvement, when there is the presence of residual tumor foci, relapsing disease, and in high-grade tumors that infiltrate vascular channels or neural parenchyma [15,16]. Intensity modulated RT (IMRT) is currently chosen for malignant SGTs’ treatment, offering protection to nerves and relative maintenance of salivary gland function [17].

The use of chemotherapeutic agents is limited and is employed as a palliative mode of treatment or in cases of advanced and/or symptomatic metastatic disease with platinum-based agents being the main option [13,15]. Other treatment options include androgen deprivation therapy (ADT) for androgen receptor positive malignant SGTs, Herceptin plus Docetaxel and ado-Trastuzumab for HER2 positive tumors as well as Entrectinib and Crizotinib in SDCs with *ETV-NTRK* fusion, and Cabozantinib in intraductal carcinoma with *NCOA4-RET* fusion [13,15].

Despite their heterogeneity, SGTs are often jointly included in clinical studies. Upon a lack of systematic SGT treatments, repetitive and metastatic tumors remain cureless on most occasions. Owing to their rareness, the genetic and epigenetic characteristics of SGTs are just emerging, and their hallmarks are not well elucidated, harnessing their therapeutic targeting. Thus, further insights on vulnerabilities and molecular landscape are necessary for these life-threatening and hard to treat tumors.

## 2. Molecular Aspects of SGT Pathology

### 2.1. Genetic Changes in SGTs

SGTs compose a diverse mosaic of various neoplasms. Their genetic profiling is currently underway, and the main genetic changes characterizing these tumors are described below. However, the elucidation of their molecular landscape remains a challenge (Table 2).

Benign BCA encompasses mutations on the *CTNNB1* gene (40%) coding for β-catenin, and part of BCAs manifest a nuclear expression of β-catenin [18]. Approximately 10% of WTs carry genetic variations, with the most common being deletions on chromosome 8, which are accompanied by deletions on 9p [19].

PA and carcinoma ex-pleomorphic adenoma (CAexPA) are characterized by translocations associated to the high-mobility group AT-hook 2 (*HMGA2*), gene which are frequent (~96%) on PAs and less common in PA and CAexPA (~30%) [20]. Moreover, *HMGA2* and pleomorphic adenoma gene 1 (*PLAG1*) fusions are exclusively occurring in PA and CAexPA and have not been detected in other SGT subtypes [21].

MECs exhibit a slow growing pattern [22], and the majority are marked by repetitive translocations, which are specific for MECs [22,23]. The CREB Regulated Transcription Coactivator 1/Mastermind Like Transcriptional Coactivator 2 (*CRTC1*/*MAML2*) fusion has a better prognostic potential [23] than *CRTC3*/*MAML2* fusion that occurs at younger ages (~35 years) [24].

AciCC exhibits activation of the EGFR/Akt/mTOR pathway [25], and a small percentage (<5%) carry rearrangements on the Myb/SANT DNA Binding Domain Containing 3 (*MSANTD3*) gene [26].

In AdCC, the fusion of the *MYB*/*NFIB* gene has been detected [27] followed by 1p36 deletion, which adds to dismal prognosis [28]. AdCC point mutations are related to *BRAF*, *KRAS*, *SPEN*, *FGFR2* and *NOTCH* signaling. Mutations of p53 occur later in the progression of high-grade AdCCs [29].

The mammary analogue secretory carcinomas (MASCs) of SGTs, likewise to breast-type, are characterized by a recurrent translocation which results in *ETV6*/*NTRK3* gene fusion [30] that is not observed in other SGTs.

The clear cell carcinoma (CCC) is exhibiting the *EWSR1*/*ATF1* gene fusion [31], which is also harbored in clear cell types of myoepithelial carcinoma, sarcoma, and odontogenic carcinoma; thus, it is possibly associated with clear cell malignancies [27].

To date, the precise molecular mechanisms underlying these malignancies remain limited. Distinct types of malignancies might express alterative genetic and epigenetic defects. The regulation of gene expression is complicated, and there are diverse mechanisms of epigenetic modifications that might interact, such as histone modifications, DNA methylation and miRNAs. A notable difference of epigenetic patterns is the fact that in contrast to genetic alterations, these changes are potentially reversible and may be restored in vivo as well as in vitro [32,33]. Therefore, the screening of distinct SGTs is essential for their diagnosis and targeted management.

**Table 2:** Characteristic/recurrent genetic aberrations on SGTs and their clinical course.

Tumor Behavior	Tumor Type	Genetic Aberration	Prevalence	Clinical Course
**Malignant**	MECs	*MECT1/MAML2* fusions	40–80%	Favorable prognosis [25]
		*CRTC3/MAML2* fusions	6%/younger ages	Favorable prognosis [24]
	AdCCs	*MYB/NFIB* gene fusions	50%	Unfavorable prognosis [25]
	AciCCs	*HTN3/MSANTD3* fusions	<5%	Indolent course [26]
		*MSANTD3* aberrations	4–15%	
	MASCs	*ETV6/NTRK3* fusions	Majority	Favorable prognosis [34]
	(Hyalinizing) CCCs	*EWSR1/ATF1* fusions	93%	Indolent course [35]
		*EWSR1* rearrangement	82%	
	Intraductal carcinoma	*NCOA4-RET*	35%	Mean OS: 5 years [36]
		*TRIM27-RET*	~10%	Aggressive/apocrine variant [36]
	Microsecretory adenocarcinoma	*MEF2C-SS18* fusion	Majority	Malignant nature [37]
	PAC and CASG	*PRKD1/2/3* translocations or mutations	*PRKD1/2/3* fusions: 13% of PACs; *PRKD1* mutations: 56–73% PACs	Indolent course [38]
			*PRKD1/2/3* fusions: 43–80% CASGs; *PRKD1* mutations: 20% CASGs	
	BCAC	*PIK3CA* mutations	~10–30%	Unfavorable prognosis [39,40]
	CAexPA	*PLAG1/HMGA2* fusions	36%	Indolent course [41]
**Benign**	PAs	*PLAG1* translocations	50%	Indolent course [42]
		*HMGA2* translocations	34–40%	
	BCA	*CTNNB1* mutations	60%	Indolent course [43]

### 2.2. Epigenetic Changes in SGTs

Epigenetics refers to inherited alterations in gene expression that can take place independently of the primary DNA sequence [44]. They are mediated by key mechanisms including DNA methylation, post-translational alterations of histones, such as acetylation/deacetylation, methylation, ubiquitination or sumoylation and the binding of microRNAs [45]. Epigenetic alterations are involved in many physiological processes such as cell differentiation and embryogenesis, but they are also implicated in altered cell states and pathological processes such as cancer [45].

Over the years and through the evolving use of different research approaches, the role of epigenetic modifications in SGTs has emerged, clarifying the landscape upon their etiopathogenetic and biologic characteristics, being associated with tumor initiation, growth, and metastatic potential (Table 3).

With respect to DNA methylation, although physiological salivary specimens lack a methylation index, benign tumors (PAs and WTs) exhibit methylation on *RASSF1*, *MGMT* and *DAPK* genes [46].

Transition to carcinoma or higher grade is an event whose molecular trigger is uncertain. The hypermethylation of *p16(INK4)* and *p14(ARF)* occurs in CAexPA and PA, whereas the hypermethylation of *RASSF1* was less prominent in PA but more evident at CAexPA [47]. Compared to non-cancerous cases, the p16 (*CDKN2a*) gene shows higher methylation rates in CAexPA [48]. *CDH1* is frequently silenced in CaexPA by promoter methylation (57%), indicating that its methylation is essential for the trigger and progression of CaexPA [49].

DNA methylation has also been detected in MEC and AdCC [50]. Specifically, *p14* hypermethylation is a hallmark of MEC progress followed by *p16*, *TP53* and *hTERT* [50]. The normal tissue lacks modifications of *p16(INK4a)*, whereas its hypermethylation was greater in higher-grade compared to lower-grade MECs [51]. *RUNX3* silencing through hypermethylation is prominent on MEC (87%), AdCC (75%) and PA (25%) compared to non-malignant specimens, adding to SGTs’ progression and poor prognosis [52]. In AdCC, the methylation of *CK* genes has been frequently detected [53,54], and a high methylation rate on *p21*, *p19*, *p18* and *p15* (69–92%) was reported [55]. In metastatic and high-grade AdCC, the hypermethylation of *RASSF1* has been detected [46].

Kanazawa et al. demonstrated that galanin receptors’ hypermethylation correlated with decreasing OS in SDCs; thus, *GALR-1/-2* gene silencing through methylation dictates a crucial epigenetic event with potentially important therapeutic and prognostic significance [56].

Wang et al. found that in *CRTC1/MAML2* fusion positive MECS, only *CLIC3* demonstrated semantic hypomethylation as well as protein overexpression; therefore, CLIC3 was suggested as an auspicious epigenetic biomarker for MECs [57]. Epigenetic E-cadherin (*CDH1*) silencing through hypermethylation is a significant pathway in MECs pathogenesis [58] as well as in AdCC [59].

Bell and colleagues found that EN1 shows constant overexpression in AdCCs [60], whereas it is positivity correlated with lymph node metastasis and poor prognosis [61], indicating the diagnostic utility of EN1 on distinct SGTs (AdCCs, PAC) [62]. Additionally, the hypermethylation of *RASSF1A* is associated with initiation and unfavorable prognosis in AdCCs [63], whereas a higher index of H3K9me3 is prognostic of brisk growth and outlying dissemination in AdCCs [64]. Through epigenetic approaches, Shao and colleagues distinguished AQP1 as an intrant oncogene concerning AdCCs; thus, its overexpression and hypomethylation suggest a signature role in AdCCs [65]. Additionally, Stratifin (14-3-3 s) is recurrently downregulated in AdCC due to hypermethylation, constituting an important mark for the evolvement of AdCC and the radiosensitization of SGTs as well [66]. Lee et al. reported that the *RARb2* and *RASSF1A* genes show regular methylation rates in distinct entities such as AciCC and AdCC [67].

In benign tumors (such as PA), histone methyltransferases (HMTs) SET08, SETDB1, and Eu are elevated compared to physiological tissue [68], further indicating that HMTs can be associated to PA differentiation. Therefore, epigenetic events affecting histones modifications are essential regulators of related genes that participate in tumor transformation [68].

Contrary to benign SGTs, malignant SGTs show low acetylation activity and therefore display a tighter chromatin format. Additionally, an inversely proportional relationship has been detected between H3 (lys9) acetylation and SGTs’ cell proliferation [69]. The methylation and hyperacetylation of H3 is linked to the aggressive phenotype of AdCC [64,70,71], and H3K9 trimethylation co-exists with high grade and invasive features of MECs. In both MECs and AdCCs, higher rates of histone H3 modifications are linked to higher cancer proliferation [71]. Particularly, the upregulation of H3K9Ac, H3K18Ac, and H3K9me3 in AdCC and upregulation of H3K9me3 in MECs were linked with increased Ki-67 expression and malignant pathological features, including aggressiveness [71]. This event is attributed to H3K9me3 and H3K9Ac due to their interaction with genes that are involved in cell proliferation (*p21^Cip1^/Notch1/ERK*) [72,73,74]. Additionally, a study demonstrated that 50% of AdCC harbor modifications in genes associated with histone alterations and chromatin remodeling (*CHD2*, *BRD2*, *ARID5B*, *KDM5A*) [75].

In the context of SGT pathogenesis and their association to epigenetic features, it has been detected that over 3600 mRNA, 280 circRNAs and 3090 lncRNAs are modified [76]. As mentioned above, *CRTC1*/*MAML2* fusion is a well-characterized tumorigenic starting event in the pathogenesis of MECs. Recently, it was reported that lncRNA/LINC00473 is a significant moderator of the *CRTC1*/*MAML2* oncogene [77]. Additionally, miR-302 is upregulated in MEC, and this event enhanced cancer cell migration in vitro [78]. To analyze the expression of apoptotic miRNAs in SGT, a study reported that miR-34a and miR-21 are overexpressed in MEC (74% and 91%, respectively). In the same study, various apoptosis-associated microRNAs (miR-5p, miR-15a, miR-16, miR-17, miR-21, miR-29, miR-34a) were upregulated in PA cases. The analysis revealed that the apoptotic modulator miR-20a was downregulated in MEC (57%) and PA (75%) [79]. These data showed that there is a modified expression of relative genes (*BAD*, *APAF1*, *BAX*, *BCL2*, *DIABLO*, *CASP8*, *TP53*) in benign PAs and in malignant MECs (*TP53*, *BAX*, *APAF1*, *DIABLO*, *BID*, *BCL2*, *CASP*-2/3/6/8) [79]. Therefore, in SGTs, modifications in apoptotic miRNAs might be tightly associated with oncogenesis. Likewise, mRNAs and lncRNAs are differentially co-expressed while interacting in PAs [80]. In particular, mRNAs are associated with events of PAs growth, and lncRNAs may affect PAs’ tumorigenesis through the modification of gene expression (IGFBP5, IGF2R and IGF2 mRNAs). Notably, this outcome shows the involvement of different epigenetic modifications on PA, taking part either individually or in combination within SGTs [80].

Moreover, in PLAG1 transgenic mice, the abnormal expression of lncRNAs and mRNAs was indicative for PAs pathogenesis through interfering with a plethora of genes, suggesting a new biomarker in PAs management [80]. The miR-17-92 cluster (miR-17/-20a) was found to be particularly upregulated and related to the aggressive biology of AdCCs, indicating a potential therapeutic target on this entity [81]. Similarly, related to MECs pathogenesis, an aberrant expression of distinct ncRNAs (ON-HSAT154433.1 upregulation/hsa_circ_00123 downregulation) could indicate prospective biomarkers and therapeutical factors on MECs [76,82].

Taken together, epigenetic alterations play a significant role in SGTs’ pathogenesis with a prognostic and diagnostic potential, enabling target therapies.

In this review, we are interested in the role of histone modifications in SGTs and particularly on histone deacetylation mechanisms that might be effective on neoplasms with relevant epigenetic signatures.

**Table 3:** Epigenetic modifications of SGTs with therapeutic/prognostic marker potential.

SG Entity	Aberration Form	Related Molecule	References
AdCCs	↑ methylation	EN1, RASSF1A, RECK, Stratifin (14-3-3 s), RUNX3	[63,66,83,84,85]
	↓ methylation	SBSN, AQP1	[65]
	↑ Promoter methylation	E-cadherin, RASSF1A, RARb2	[54,59,67,86]
	Histone trimethylation	H3K9me3	[64,87]
	↑ acetylation	H3K9Ac, H3K18Ac	[71,88]
	miR-17-92 ↑regulation	miR-17/-20a	[81]
MECs	Aberrant expression of ncRNAs	NONHSAT154433.1, has-circ-0012342	[76,82]
	↑ methylation	RUNX3, H3K9Me3	[52,85,88]
	↑ acetylation	H3K9Ac, H3K18Ac	[71,88]
SDCs	↑ methylation	GALR-1/-2	[56]
	↑ Promoter methylation	RASSF1A, RARb2,	[67]
PAs	Dysregulated expression (PLAG1 gene related)	9110 lncRNAs/7750 mRNAs	[80]
CAexPA	↑ Promoter methylation	CDH1 (E-cadherin)	[49,89]
ACCs	↑ Promoter methylation	RASSF1A, RARb2	[67]

↑ refers to increased, ↓ refers to decreased.

## 3. Role of Histone Deacetylation in SGTs

### 3.1. Biochemical and Functional Aspects of Histone Acetylation/Deacetylation

**Figure 1:**
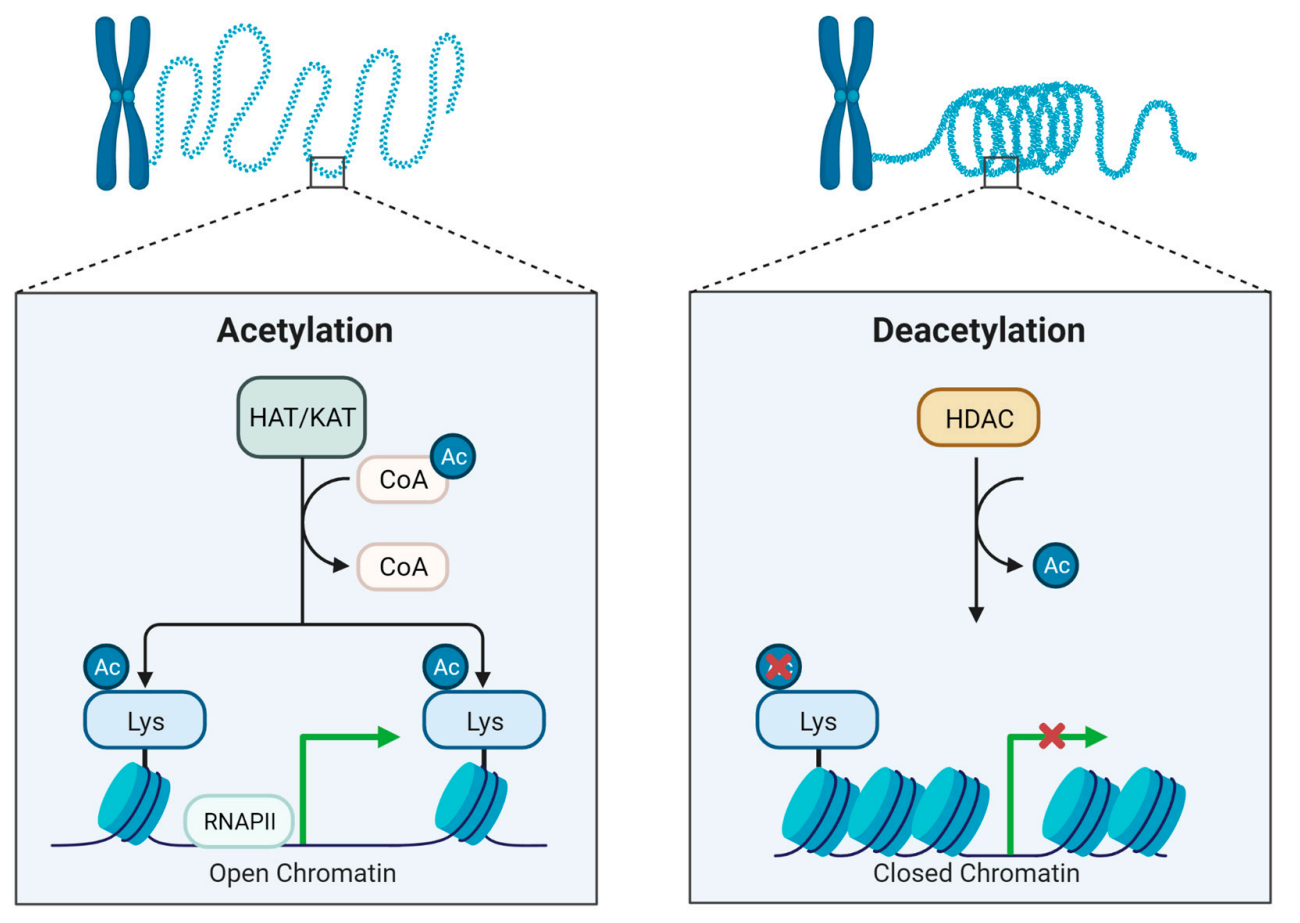
The processes of histone acetylation and deacetylation are fundamental mechanisms of epigenetic regulation, controlling gene expression. Histone acetyltransferases (HATs/KATs) catalyze the addition of acetyl groups to lysine residues of histone proteins, which promotes the relaxation of chromatin structure and enhances accessibility of DNA to transcription factors. Conversely, histone deacetylases (HDACs) remove acetyl groups from histones, leading to chromatin compaction and repression of gene transcription. This figure was created using the tools provided by BioRender.com (accessed on 3 May 2023).

The abundance of lysine residues in histones creates an ideal environment for modifications, especially acetylation and its opposite reaction, deacetylation (Figure 1). The former occurs when an acetyl group from an acyl-CoA donor is placed in the ε-amino group of the lysine side chain via the action of specialized enzyme called histone acetyltransferases (HATs) or recently renamed as lysine acetyltransferases (KATs) since acetylation has been also discovered in non-histone proteins (6). By adding acetyl residues, they may neutralize the positive charge of lysine residues eventually “loosening” the bond of histones with DNA, allowing recruitment and binding of transcriptional factors. There are more than 20 types of HATs/KATs that are freely distributed either in the nucleus or the cytoplasm, acting as transcriptional co-activators [90].

Both acetylation and deacetylation are highly reversible processes, thus for effective gene regulation, there is necessity for balance between the addition and the removal of acetyl groups [90,91]. Removal of acetyl groups (deacetylation) is achieved by histone deacetylases (HDACs) or lysine deacetylases (KDACs) [90]. HDACs function in “re-packing” tightly the DNA with histones via positively charging the lysine residues inducing a close chromatin complex and blocking RNA polymerases from initiating DNA transcription [92].

To this day, 18 HDACs have been identified. The interaction with several co-factors and the existence of a deacetylase domain are the two main characteristics taken into account for the subcategorization of HDACs in two large families: the deacetylase family which comprises of zinc-dependent enzymes and is furtherly subclassified in type-I (HDAC1, -2, -3, -8), type-II (HDAC4, -5, -6, -7, -9, -10) and type-IV (HDAC11), with type-II consisting of two subfamilies of type-II^a^ and type-II^b^ due to differences in their domain composition [92].

Each class is further divided into subtypes, and each HDAC subtype has distinct cellular locations and substrate specificities. These enzymes are involved in various cellular processes, such as gene expression regulation, cell cycle progression, apoptosis, and cellular differentiation. The following table summarizes HDAC classes, subtypes, cellular locations, substrate specificities, and main functions (Table 4).

Studies investigating HDACs expression and activity in carcinogenesis are progressively increasing [91,95]. More specifically, Type I HDACs are often dysregulated in cancer regulating cell proliferation and apoptosis [96]. Regarding class-II^a^ HDACs, HDAC4 may exhibit either a tumor suppressive or oncogenic role [97] whereas HDAC5 and HDAC9 play a significant role in medulloblastomas [98]. In lung cancer, HDAC6 has been linked to epithelial-mesenchymal transition (EMT) via the SMAD3 pathway [99] and HDAC10 was shown to induce proliferation via the AKT signaling pathway [100].

SIRT1 has been upregulated in prostate cancer and leukemia [101,102] while SIRT2 was shown to possess tumor suppressive properties [103]. In urothelial cancer, increased SIRT3 levels were linked to cancer cell cycle arrest. Increased SIRT3 and SIRT6 levels have been detected in breast cancer [103,104] and reduced in gliomas as well as in ovarian cancer [105].

### 3.2. Role of HDACs in Different SGT Types

HDAC family members have been detected in various types of malignancies such as oral cavity cancer, but the SGTs remain an entity which has not been thoroughly investigated [106].

Based on the Human Protein Atlas (HPA), HDACs have moderate to increased protein-expression levels in breast, cervical, colon, endometrial, liver, lung, ovarian, pancreatic, prostate, renal, glioma, lymphoma, and HN cancer [107]. In HN malignancies, HDAC class-I and -II expressions vary (Table 5). The majority of HDACs members are expressed to almost 100% (HDAC2, -9, -10, SIRT3, -5, -6, -7), ~75% (HDAC1, SIRT1) and ~25% (HDAC3, -6). HDAC5 is not included in the above due to a lack of expression [107]. Almost 39% of NUT midline carcinomas (NMCs) are located in HN [108]. In the case of NMC present in the parotid gland, immunohistochemical analysis showed strong to moderate immuno-expression of HDAC2, -4 and -6, and phospho-HDAC4, -5, -7, in the neoplastic cells [109].

Moreover, an immunohistochemical study from our group evaluated the expression of HDACs (-1, -2, -4 and -6) in SGTs [110]. HDAC subtypes were abundantly expressed in both benign and malignant samples. In malignant tumors, HDAC1, 2, 4 and HDAC6 expression was observed in 14%, 82%, 36% and 18%, respectively. High HDAC expression was present in 25% to 89% of HDAC-positive SGTs and was not associated with patients’ age and gender. HDAC2 expression was suggested as a positive prognostic factor and was associated to extended overall survival (OS) on high-grade malignant SGTs. HDAC6 overexpression, on the other hand, might have a negative impact on the OS of high-grade SGT cases given that HDAC6 knockdown was linked to extended OS [110].

Wagner et al. studied the immunohistochemical expression of acetylated histone H3 and Ki67 in 84 cases of SGTs with tissue microarrays (TMAs). The analysis showed that aggressive SGTs were under-acetylated, in contrast to benign SGTs, leading to chromatin condensation. Moreover, H3 was oppositely associated with Ki67 expression on SGTs, indicating that HDACs increase cell proliferation [69].

Chidamide (HBI-8000/Tucidinostat), which induces H3 and H4 acetylation, has shown a selective inhibitory effect to HDACs (class-I and -II) [111]; another study reported on the efficacious stability/durability and response of a patient with advanced AdCC during a phase-I trial [112]. This was the case for the preclinical administration of Chidamide in AdCC that exhibited significant inhibition and satisfactory results, suggesting its potential effectiveness for AdCC patients [113].

Preclinically, HDAC6i/KA2507 showed antitumor efficacy in 93 human cancer cell lines and in in vivo cancer models, while its antitumor efficiency was increased upon the co-administration of a checkpoint inhibitor and subsequently allocated to the first clinical study where SGTs (AdCC, SDCs) were also included [114]. Based on prior NGS (next-generation sequencing) analysis in AdCC [75,115] and the supporting radiological/clinical findings of two salivary AdCC patients in a clinical study of vorinostat evaluation in advanced malignancies [116], vorinostat was further evaluated in a phase-II trial of AdCCs with promising results [117].

Studies using in vitro and in vivo models demonstrate the synergistic effect of different mechanisms such are checkpoint and HDAC inhibitors [118]. Several trial data are also encouraging upon solid and blood malignancies [119]. The Keynote-028 study depicted 3/26 SGT respondents with PDL-1 expression (>1%) [120]. A phase-II/vorinostat study in 30 relapsed/metastatic AdCCs reported 97% clinical advantage and 2 impartial responses [121]. In this frame, Rodrigues et al., performed a phase-II study that investigated the combinatory effect of pan-HDACi/vorinostat together with the anti-PD1i/Pembrolizumab in different SGTs, reporting important clinical outcomes in different types (lymphoepithelioma-like/AciCC/AdCC carcinomas) [122]. Τhe underlying mechanisms and types of inhibition in the various subtypes of SGTs are discussed in detail in the following section.

The integration of complementary bioinformatics approaches is crucial for the comprehensive identification and analysis of HDAC expression in a variety of SGT types, providing insights into their functional roles, molecular mechanisms, and potential as therapeutic targets. Leveraging computational methods and tools, such as artificial intelligence (AI) and machine learning (ML), researchers can obtain crucial insights into the roles of HDACs in SGT biology and explore their potential as therapeutic targets. Additionally, in silico analyses of publicly available datasets, such as The Cancer Genome Atlas (TCGA) [123] or the Gene Expression Omnibus (GEO) [124], can provide a wealth of information on HDAC expression patterns as well as their association with other proteins and molecular pathways.

Gene expression profiling techniques, such as microarrays, RNA-Seq, and single-cell RNA-sequencing (scRNA-Seq), enable the assessment of global gene expression levels, including HDACs, across various tumor types [124]. ScRNA-Seq, in particular, provides a higher resolution of the cellular heterogeneity within SGTs, allowing for the identification of HDAC expression patterns in specific cell populations [125]. By comparing the expression profiles between tumor and normal tissues or among distinct tumor types, scientists can identify differentially expressed HDACs that may contribute to tumor development or progression [126]. Furthermore, co-expression analysis can help identify genes that are co-expressed with HDACs in SGTs, potentially uncovering functionally related or co-regulated genes and providing insights into the molecular mechanisms of HDACs in these tumors [127]. Further downstream, gene set enrichment analysis (GSEA, also called functional enrichment analysis) can be employed to delineate the biological processes and molecular pathways associated with differentially expressed HDACs, offering a deeper understanding of their roles in SGTs [128]. Protein–protein interaction network (PPIN) analysis can explore the molecular interactions between HDACs and other proteins in SGTs, potentially revealing novel therapeutic targets or emphasizing the role of HDACs in the context of other relevant proteins [129].

Lastly, AI and ML techniques can be applied to classify SGT types based on HDAC expression patterns or predict patient outcomes [130]. By incorporating multi-omics data, such as genomics, transcriptomics, and proteomics, ML algorithms such as support vector machines (SVMs), random forests (RF), or deep learning (DL) models can offer a more comprehensive understanding of the molecular landscape of SGTs, facilitating the identification of potential biomarkers for diagnostic or prognostic purposes and aiding in the development of personalized therapeutic strategies [131].

## 4. Targeting Options of HDACis in SGTs

### 4.1. HDAC Inhibition

HDACis are natural or semi-factitial derivatives of natural products [10]. Based on their structure, they are classified into small-chain fatty acids such as valproic acid (VPA) and butyrate acid, cyclic peptides/depsipeptide-FK228 (Romidepsin), hydroxamic acids (SAHA), LBH589 (Panobinostat), TSA (Trichostatin A), AR-42 (a hydroxamate-phenylbutyrate derivative), cyclic tetra-peptide (Trapoxin A) and benzamides (Entinostat/MS-275 and MGCD0103) [11]. Lately, the role of synthetic alkyl 4HR as a new HDACi has emerged [132,133]. Additionally, tumor angiogenesis is inhibited by a variety of actions that the hydroxamic derivative LBH589 has on endothelial cells [134].

HDACis might promote oncogenic mechanisms through various gene signaling pathways. SAHA acetylates tumor suppressors such *p53* and miR-15/16, and it favors the alteration of mutant into wild-type p53 cancer cells [135]. MPT0G009 administration enhances TRAIL expression [136], and valproic acid prompts the activation of *p21* and *PARP* [137]. Likewise, HDACis lead to histone refracturing on the p21 domain, modulate the cell cycle genes (*Cdk-1*, *Cdk-4*, *Cdk-6*) expression, and regulate serine/threonine kinases [136,137].

HDACis might also affect several biological processes in cancer cells such as apoptotic activity by balancing gene expression via histone methylation or/and acetylation as well as by acetylation of diverse non-histone proteins such as STAT-3, p53 and tubulin. Additionally, HDACis can regulate apoptosis by inducing ubiquitin *(UBB)* gene expression or *Mcl-1* and *Bcl-2* [138], modulate caspase proteins by inducing TRAIL expression, downregulate c-FLIP factors and regulate cell-cycle arrest at active or pathologically proliferative malignant cells [139,140,141].

Over the years, SGTs’ treatment relies on combination therapy with platinum, single-agent, HER-2, EGFR, c-kit, tyrosine kinase and microtubular inhibitors, fusion transcripts targets, hormonal or combination therapy with biologics and immune checkpoint inhibitors [142]. Immunotherapy in combination with other agents for SGTs such as HDAC inhibitors is a field that is now beginning to be explored (Table 6).

#### 4.1.1. HDACis in Mucoepidermoid Carcinoma (MEC)

Unlike combination therapies, multi-target agents are in favor of more predictable pharmacokinetics and may reduce therapeutical cross-reactions [150]. CUDC-101 is a molecule composed of combined EGFR and HDAC inhibitors that demonstrates efficacy in comparison to combined therapies including EGFRi (Erlotinib) and HDACis (SAHA). This agent is adequate toward almost 40 various cell lines among 16 types of cancers (in vitro/in vivo) [151,152] and shows efficacious tolerance on clinical studies [153]. CUDC-101 might blunt MEC cancer stem-cell tumorigenicity, shows irrevocable and robust cytotoxicity on MEC cells and affects apoptosis through caspase 3/7 activation [147]. As proposed by Sharma and colleagues, together, HDACi and EGFRi suggest a promising agent against MEC tumors. Additionally, CUDC-101 is effective in simultaneously downregulating p-EGFR and upregulating histone acetylation (H3K9ac and H4K12ac) compared to EGFR and SAHA. Therefore, coupling EGFRi and HDACi might bring up to fivefold cell cytotoxicity compared to EGFR or HDAC single treatment [147].

HDAC7 modulates cell proliferation, differentiation, apoptosis, and migration in physiological and pathological conditions [154]. The aberrant expression of HDAC7 has been observed in gastric cancer [155], breast cancer [156], leukemia and lymphoma [157], glioma cancer [158], lung cancer [159], ovarian cancer [160] and nasopharyngeal carcinoma [161]. The broad expression of HDAC7 is related to poor prognosis and metastasis [155,159]. In addition, various HDACis such as Trichostatin A [162], valproic acid [163] and vorinostat [164] target HDAC7. The role and the underlying mechanism of HDAC7 in MEC is yet to be clarified. Ahn and colleagues studied the association between HDAC7 and autophagy in cancer cells [144]. YD-15 and Mc3 cells were treated with distinct HDAC7 siRNAs. HDAC silencing induced autophagy in MEC cells, by regulating LC3-II and p62 expression, possibly through a pro-survival mechanism which resembles the apicidin-induced autophagy. Moreover, HDAC7 suppression affected apoptosis through activating caspases [144]. Additionally, MAPK (mitogen-activated protein kinase) activity is associated with a reduction in ERK phosphorylation, hypothesizing that HDAC7 silencing allies with ERK inactivation in MEC. Interestingly, HDAC7 might play a robust oncogenic role in MEC cells by inhibiting cell proliferation (G2/M arrest), downregulating c-Myc and upregulating p27 accumulation [144].

Apicidin might inhibit the proliferative potential of MEC cells [143]. Apicidin-prompted apoptosis and autophagy might act in a competitive way; therefore, autophagy may act protectively on trigger apicidin-prompted apoptosis in MEC. Additionally, apicidin induces an apoptotic effect by inactivating ERK and AKT/mTOR signaling and activating JNK (c-Jun NH_2_-terminal kinase). The autophagic response of apicidin is also mediated by inactivating AKT/mTOR pathways. Apicidin cytotoxicity is limited by JNK inhibition and enhanced by AKT and ERK inhibition. These events are probably caused by the downregulation of IGF1R, which upregulates the AKT/mTOR and MAPK signaling pathways [143].

Sodium butyrate or NaBu refers to an apoptotic HDACi [165]. Τhe route which NaBu uses in order to implement its apoptotic activity in MEC cells is unclear. Survivin (BIRC5) is an adaptor protein that affects mitosis and apoptotic inhibition [166]. Survivin’s function depends on its cellular location [167,168]. In SGTs, when expressed in cytoplasm, it is associated with poor prognosis [165]. In the nucleus of malignant cells, survivin binds with STAT3, thus inhibiting the transcriptional potential of downstream factors of the STAT3 pathway [148,169,170]. In oral YD15 and MC3-MEC cell lines, NaBu might decrease cell viability, inhibit the transcriptional potency of the Bcl-XL/STAT3 molecule, and enhance the cleavage of caspase 9 and caspase 3. In addition, NaBu acetylates survivin, causing its nuclear translocation, which in turn represses STAT3 oncogenic activity and prompts caspase-reliant apoptosis in MEC [148]. In vivo, NaBu might attenuate tumor proliferation without hepatic or renal toxicity [148].

#### 4.1.2. HDACis in Adenoid Cystic Carcinoma (AdCC)

AdCC is an infrequent tumor often occurring in salivary glands. Almost half (35–50%) of AdCC patients bring gene mutations associated with the remodeling of chromatin and histone alterations [75,113,115]. Hitherto, there is no targeted agent approved by the FDA for its treatment [117]. In in vivo as well as in vitro trials, vorinostat usage minimized the CSCs rate [146]. Despite the total depletion in tumor cell viability, the prolonged administration of vorinostat led to CSCs proliferation in AdCC primary cells. Nevertheless, synergistically, cisplatin along with vorinostat decreased the viability of HACC2A as well as HACC6 tumor cells by 73.5% and 77.5%, respectively. Vorinostat stimulated cancer cells’ sensitization in cisplatin through a mechanism that couples CSCs depletion and triggering cell senescence [146].

Chidamide is a new HDACi that falls into the benzamide family. Specifically, it increases the acetylation potential in the H3 histone, is more stable than SAHA and eclectically intercepts the effect of HDAC1, -2, -3 and -10 [171]. To date, it has been only authorized by China’s FDA as a choice over relapsed or refractory PTCL management [172]. Chidamide implements its antitumor effect through various mechanisms. Additionally, it might reverse the EMT and treatment resistance of neoplastic cells [171], induce apoptosis and tumor cell growth arrest [173] and amplify apoptotic activity as well as platinum-based DNA-damage response in non-small cell lung cancer (NSCLC) [174]. In phase-I and -II studies, chidamide has shown favorable pharmacokinetics and pharmaco-dynamics, notable tolerability, and antitumor efficiency [112,175].

In preclinical study, chidamide and cisplatin (CDDP) restrained the proliferation of AdCC xenografts [113]. In AdCC-2 and AdCC-3 cells, the cleavage of caspase 3 was not observed after chidamide administration, whilst the chidamide-induced apoptotic path is still unclear [113]. Chidamide may also inhibit cell development and proliferation through a dosage and time-dependent mode and affect the G2/M phase of cells. Moreover, it can decrease p-AKT expression and upregulate Ac-H3 protein expression in AdCC. Thus, chidamide inhibits cell growth by increasing H3 acetylation and intervening on AKT phosphorylation and G2/M cell-cycle arrest on AdCC cells [113].

#### 4.1.3. HDACis in Salivary Gland Ductal Adenocarcinoma (SGDA)

**Figure 2:**
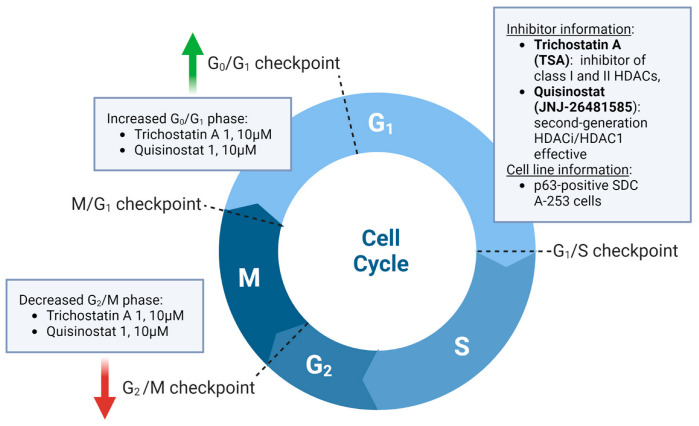
Effects of HDAC inhibitors TSA and Quisinostat on the cell cycle of salivary gland ductal adenocarcinomas (SGDAs). This figure is based on the work of Nakano et al. [149] and was created using the tools provided by BioRender.com (accessed on 2 May 2023).

The p53 family member p63 maintains two characteristic isoforms, which are crucial for proliferating and differentiating epithelial cells. TAp63 disposes a pro-apoptotic effect, whereas DNp63 is oncogenic [176]. p63 is upregulated in various malignancies such as SGTs [177]. Diagnostically, DNp63 (p63/p40) applies for histologically differentiating SGTs [178]. So far, the roles and mechanisms of p63 on SGTs are not well known. Nakano and colleagues transfected A253 (SGDA) cells with siRNA-p63 or injected with an HDACi (Trichostatin A and Quisinostat) [149] (Figure 2). As shown, the knockdown of *p63* with siRNA-p63 may induce tight junction (TJ) proteins, differentiation markers and aberrant cell metabolism in human SGDA A253 cells. Both p63 inhibition and HDACis hinder the proliferation of A253 cells. In addition, HDACis may inhibit p63 protein expression, enhance TJ proteins expression and prevent cell migration in SGDA cells, suggesting their potential therapeutic utility [149].

### 4.2. Phase-I Clinical Studies of HDACis in SGTs

#### 4.2.1. Vorinostat in a Phase I Study

Vorinostat inhibits class-I as well as class-II HDACs and holds FDA approval for administration at refractory CTCL [179]. In phase-I trials, vorinostat was administered on patients with hepatic malfunction and solid malignancies [116]. Before entering the trial, all AdCC patients had disease advancement in the former treatment. A salivary AdCC patients’ response to treatment led to the enrollment of a further four patients with AdCC. Additionally, one patient with AdCC had a partial response, and four reached disease steadiness in a period of 4, 12, 12 and 13 months [116].

#### 4.2.2. KA2507 Selective HDAC6 Inhibitor

Ubiquitously expressed HDAC6 is located on the cytoplasm [180] and affects tumor cell modifications by inducing anchorage-independent proliferation in transmuted cells [181]. Via post-translational modifications of its substrates and cytoplasmic-interacting molecules (such as α-tubulin, cortactin and HSP90), HDAC6 affects a variety of cellular processes such as intracellular signaling, DNA-damage response, gene transcription, and cell movement. Disruption of the physiological expression and function of HDAC6 affects tumorigenic modifications, cancer cell growth, mitosis, invasion, and migration [182]. So far, there is no FDA approval for HDAC6 inhibitors except for the pan-HDACis such as LBH589, PXD101, Romidepsin and SAHA [183,184].

KA2507 is hydroxamic acid family inhibitor of nano-molecular weight that eclectically targets HDAC6. From 2017 to 2019, in a phase-I trial, KA2507 was administrated in 20 patients with solid tumors including heavily pretreated, treatment-refractory patients [114]. Patients with salivary AdCC (15%) and SDC (5%) were included. Administrating KA2507 had positive tolerance and showed no evidence of mechanism-based toxicity (myelosuppression, cardiac toxicity). Stable disease was observed in a patient with AdCC.

In vitro, KA2507 lacks inhibition potency on proliferative malignant cells (human/mouse). On the other hand, KA2507 demonstrated efficacy in in vivo preclinical cancer types [114]. The selective inhibition of HDAC6, barren of off-target class-I HDAC effect, correlated with increased α-tubulin acetylation and the downregulation of histone H3 acetylation. Moreover, in mouse models, KA2507 can modulate antitumor response through the alteration of several immune biomarkers. It was shown to reduce STAT3 activation, decrease the expression of PD-L1, increase type-I MHC proteins and show efficacy when co-administered with a checkpoint inhibitor [114].

### 4.3. Phase-II Trials of HDACis in SGTs

#### 4.3.1. SAHA Treatment in AdCC

AdCC is an uncommon type of malignant SGT, encompassing 1% of HN cancers and 5–10% of the malignant SGTs [185]. Surgical intervention is the preferred way of management toward localized AdCC arising from major and minor salivary glands with postoperative radiotherapy to optimize local disease control. AdCC has a primary slow-moving profile but increased migration likelihood. In metastatic stages, AdCC shows low responsiveness to available therapies [186].

In a multi-institutional clinical trial (phase-II) of SAHA administration, 30 patients with progressed or metastatic AdCC were included [117], exhibiting in most cases (20) decreased tumor size. In addition, patients exhibited a median stable disease (8 months), progression-free survival (10 months) and overall survival (11.5 months). Partial response was noted in two cases, whilst three patients withdrew due to toxicity. Toxicities (grade-III) included lymphopenia (23%), hypertension (10%), fatigue (10%), oral achiness, cephalalgy and thromboembolic incidents. Moreover, two AdCC patients who underwent vorinostat therapy manifested early (after 2–3 cycles) clinical improvement including pain and tiredness reduction, weight advantage, radiological improvement, reduced liver lesion and normalization of liver enzymes. The late responses were attributed to vorinostat immunostimulation, which possibly requires prolonged time to manifest when compared to other therapeutic agents [117].

#### 4.3.2. Combination of Pembrolizumab and Vorinostat in SGTs

Despite the encouraging response in HER2-positive SDC treatment [187], most SGT trials are rare, have a number of limitations and have low success rates. In a phase-II clinical trial (2015–2017), vorinostat and Pembrolizumab were administrated in recurring/metastatic SCCHN (squamous cell carcinomas of head and neck of HN/25 patients) together with SGTs (25 patients) [122]. Stable disease was met in 14 patients (56%), while responses were prolonged and observed in SGTs (16%) including a lymphoepithelioma-like parotid carcinoma, an AdCC and two acinic cell carcinoma patients. Toxicities were broader than Pembrolizumab alone, and adverse events were observed in 13 SGT patients (52%). Moreover, vorinostat-related dose reductions included nine patients (36%). Vorinostat-related toxicity included fatigue and weight loss (1), malaise (1), severe fatigue (2) and increased serum creatinine (5). The response was promising, although the dual treatment can exhibit lower responses in SGTs than HN [122]. A possible interpretation of the lower response rate in SGTs is their reduced rates of PD-L1 expression, reduced tumor mutational profile and non-association to previous tobacco usage, alcohol, or viral exposure.

## 5. Conclusions—Outlook

Malignant cells exploit epigenetic modifications in order to regulate tumorigenic proliferation, migration, and drug effectiveness [33,36]. Accordingly, curative agents tackling epigenetic mechanisms may be novel frontiers in cancer therapy. HDACis induce chromatin acetylation, but the molecular pathways that determine HDACis efficiency as sensitizing agents remain elusive. Alone or in synergy with known therapies, HDACis may inhibit resistance to antitumor treatment.

In vitro, EGFRi and HDACi (CUDC-101) display synergistic effects on MEC cells. Additionally, the inhibition of AKT and ERK amplifies apicidin-related cytotoxicity in MEC cells. Vorinostat and cisplatin synergistically reduce the viability of ACC cells. Vorinostat boosts cancer cells’ sensitization to cisplatin, depletes CSCs, and induces the activation of cellular senescence. Chidamide in synergy with cisplatin can hinder the growth of AdCC cells. HDACis, such as Quisinostat and TSA, may enhance TJ protein expression, downregulate p63 expression, and prevent cell migration in SGDA cells. Moreover, in an in vivo preclinical model, KA2507’s effectiveness was boosted by the co-administration of an immune checkpoint inhibitor. Clinically, HDAC6-selective inhibitor KA2507 is well tolerated with no significant toxicities. Therefore, KA2507 optimal use may be preferable in a combinatorial immunotherapy approach.

In a phase-I trial, vorinostat treatment of AdCC cells showed partial response and disease stabilization. In a phase-II study with vorinostat, most patients showed disease stability and decreased tumor size. HDAC7 inhibition triggers the apoptotic cell death and autophagy in MEC cells. In addition, NaBu can cause the nuclear translocation of survivin, inhibit growth, and induce apoptosis in MEC cells. HDAC2 is related to extended OS on high-grade SGT tumors. Conversely, HDAC6 upregulation is negatively correlated with the OS of high-grade malignant SGT patients, suggesting that HDAC6 inhibition holds promise in SGT treatment. Therefore, HDACis may be potent agents that exhibit antitumor activities.

Conclusively, SGT trials are currently infrequent and may have a number of limitations as well as low success rates. Nevertheless, the continuous investigation of molecular targets holds the key to successful cancer treatments, and a detailed profiling of HDAC modifications along with their inhibition mechanisms might prove to be a promising approach toward a more effective SGT treatment.

## Figures and Tables

**Table 1:** Salivary gland neoplasms according to WHO 2022.

Salivary Carcinomas Not Otherwise Specified (NOS) and Emerging Entities	Malignant Epithelial Tumors
Large-cell neuroendocrine carcinoma	Acinic cell carcinoma
Lymphoepithelial carcinoma	Adenoid cystic carcinoma
Oncocytic carcinoma	Basal cell adenocarcinoma
Small-cell neuroendocrine carcinoma	Carcinoma ex pleomorphic adenoma
Squamous cell carcinoma	Carcinosarcoma
Undifferentiated carcinoma	Clear cell carcinoma
**Benign Epithelial Tumors**	Epithelial–myoepithelial carcinoma
Basal cell adenoma	Intraductal carcinoma
Cystadenoma	Microsecretory adenocarcinoma *
Ductal papillomas	Mucinous adenocarcinoma *
Intercalated duct adenoma *	Mucoepidermoid carcinoma
Keratocystoma *	Myoepithelial carcinoma
Lymphadenoma	Polymorphous adenocarcinoma
Myoepithelioma	Salivary duct carcinoma
Oncocytoma	Sclerosing microcystic adenocarcinoma *
Pleomorphic adenoma	Sebaceous adenocarcinoma
Sclerosing polycystic adenoma *	Secretory carcinoma
Sialadenoma papilliferum	**Uncertain Malignant Potentiality**
Striated duct adenoma *	Sialoblastoma
Warthin tumor	

* New entities included in the newest WHO classification.

**Table 4:** HDAC classes, subtypes, cellular locations, substrate specificities, and functions.

HDAC Class	Subtypes	Cellular Location	Substrate Specificity	Functions and Targets
Class-I	HDAC1, HDAC2, HDAC3, HDAC8	Nucleus	All four core histones (HDAC1,2); H3, H4 (HDAC8)	Gene repression; Cell-cycle regulation; Apoptosis; DNA repair [93]
Class-II^a^	HDAC4, HDAC5, HDAC7, HDAC9	Nucleus and Cytoplasm	All four core histones (HDAC4,5)	Cell differentiation; Apoptosis; Angiogenesis; Response to external signals [94]
Class-II^b^	HDAC6, HDAC10	Cytoplasm (HDAC6); Nucleus (HDAC10)	H3K9, H3K56, α-tubulin (HDAC6); Polyamine catabolism (HDAC10)	Regulation of microtubule stability; Aggresome formation [94]
Class-III	SIRT1, SIRT2	Nucleus and Cytoplasm	H4K16, H3K9 (SIRT1); H4K16, H3K56 (SIRT2)	Aging; Metabolism; DNA repair; Stress resistance [93]
	SIRT3, SIRT4, SIRT5	Mitochondria	H4K16 (SIRT3); Mitochondrial metabolism (SIRT4,5)	Energy homeostasis; Oxidative stress response [93]
	SIRT6, SIRT7	Nucleus (SIRT6); Nucleolus (SIRT7)	H3K9, H3K56 (SIRT6); H3K18 (SIRT7)	Genome stability; Ribosome biogenesis [93]
Class-IV	HDAC11	Nucleus and Cytoplasm	H3K9, H3K14	DNA replication regulation; Immune regulation [93]

**Table 5:** HDAC expression in HN malignancies and SGTs.

Categorization	Study Type/Method	HDAC Type	Expression Rate	Reference
HN malignancies	Tissue sections (FFPE)/IHC	HDAC2, -9, -10	~100%	[107]
		SIRT3, -5, -6, -7	~100%	[107]
		HDAC1, SIRT1	~75%	[107]
SGTs	Tissue sections (FFPE)/IHC	HDAC1	Benign SGTs: 30%	[110]
			Malignant SGTs: 14%	[110]
	Tissue sections (FFPE)/IHC	HDAC2	Benign SGTs: 86%	[110]
			Malignant SGTs: 82%	[110]
	Tissue sections (FFPE)/IHC	HDAC4	Benign SGTs: 44%	[110]
			Malignant SGTs: 36%	[110]
	Tissue sections (FFPE)/IHC	HDAC6	Benign SGTs: 11%	[110]
			Malignant SGTs: 18%	[110]

**Table 6:** Research studies evaluating HDACi efficacy in salivary gland malignancies.

Type of Salivary Gland Malignancy	Substance	Main Outcome	Reference
MEC (cell line)	Apicidin (HDACi)	↓ proliferative potential	[143,144]
MEC (cell line)	Vorinostat (HDACi)	↓ cancer stem cell population	[145]
	Vorinostat (HDACi) + cisplatin	↑ sensitivity to cisplatin	
AdCC (cell line) Mice transplanted with tumor cells	Vorinostat (HDACi)	↓ population of cancer cells and stem cells	[146]
	Vorinostat (HDACi) + cisplatin	Further ↓ cancer stem cell population	
AdCC (cell line)	Chidamide (HDACi)	↓ proliferative potential	[113]
	Chidamide (HDACi) + cisplatin	Cell cycle arrest	
MEC (cell line)	Vorinostat (HDACi)	↓ cancer stem cell population	[69]
	Vorinostat (HDACi) + Εmetin (anti-NF-κB)	Achieving combined therapeutic effect	
MEC (cell line)	CUDC-101 (EGFRi + HDACi)	↑ cytotoxicity ↓ cancer stem cell oncogenicity	[147]
MEC (cell line) Mice transplanted with tumor cells	Sodium butyrate (HDACi)	↓ proliferative potential no organ toxicities in vivo	[148]
SGDA (cell line)	HDACi (TSA + Quisinostat)	↓ proliferation and migration potential ↑ epithelial barrier function	[149]

↑ refers to increased, ↓ refers to decreased.

## Data Availability

Not applicable.

## Abbreviations

AciCC: acinic cell carcinoma; AC-NOS: adenocarcinomas non-otherwise specified; AdCC: adenoid cystic carcinoma; ADT: androgen deprivation therapy; AI: artificial intelligence; BCA: basal cell adenoma; BCAC: basal cell adenocarcinoma; CAexPA: carcinoma ex-pleomorphic adenoma; CASG: cribriform adenocarcinoma of salivary gland; CCC: clear cell carcinoma; CDDP: Cisplatin; CTCL: cutaneous T-cell lymphoma; DL: deep learning; DHS: deoxyhypusine synthase; EMT: epithelial-mesenchymal transition; GEO: gene expression omnibus; GSEA: gene set enrichment analysis; HAT: histone acetyltransferase; HDAC: histone deacetylase; HDACi: histone deacetylase inhibitor; HMT: histone methyltransferase; HN: head and neck tumor; HPA: human protein atlas; IMRT: intensity modulated RT; JNK: c-Jun NH2-terminal kinase; KAT: lysine acetyltransferase; KDAC: lysine deacetylase; MAPK: mitogen-activated protein kinase; MASC: mammary analogue secretory carcinoma; MEC: mucoepidermoid carcinoma; ML: machine learning; MRI: magnetic resonance imaging; MYE: myoepithelioma; NAD: nicotinamide adenine dinucleotide; NGS: next-generation sequencing; NMC: NUT midline carcinoma; NSCLC: non-small cell lung cancer; NTD: N-terminus domain; OS: overall survival; PA: pleomorphic adenoma; PAC: polymorphous adenocarcinoma; PPIN: protein–protein interaction network; PTCL: peripheral T-cell lymphoma; RF: random forests; SCCHN: squamous cell carcinomas of head and neck; SAHA: vorinostat; scRNA-Seq: single-cell RNA sequencing; SDC: salivary duct carcinoma; SGDA: salivary gland ductal adenocarcinoma; SGT: salivary gland tumor; SVMs: support vector machines; TCGA: The Cancer Genome Atlas; TJ: tight junction; TMA: tissue microarray; TSA: trichostatin A; UBB: ubiquitin gene; VPA: valproic acid; WT: Warthin’s.
